# The Spectrum of Cutaneous Manifestations in Lupus Erythematosus: A Comprehensive Review

**DOI:** 10.3390/jcm13082419

**Published:** 2024-04-21

**Authors:** Aleksandra Fijałkowska, Marcelina Kądziela, Agnieszka Żebrowska

**Affiliations:** Department of Dermatology and Venereology, Medical University of Lodz, Haller sq. 1, 90-647 Lodz, Poland; aleksandra.fijalkowska@stud.umed.lodz.pl (A.F.); marcelina.kadziela@stud.umed.lodz.pl (M.K.)

**Keywords:** cutaneous lupus erythematosus, systemic lupus erythematosus, skin manifestations, autoimmunity

## Abstract

Lupus erythematosus (LE) is an autoimmune inflammatory disease with complex etiology. LE may present as a systemic disorder affecting multiple organs or be limited solely to the skin. Cutaneous LE (CLE) manifests with a wide range of skin lesions divided into acute, subacute and chronic subtypes. Despite classic forms of CLE, such as malar rash or discoid LE, little-known variants may occur, for instance hypertrophic LE, chilblain LE and lupus panniculitis. There are also numerous non-specific manifestations including vascular abnormalities, alopecia, pigmentation and nail abnormalities or rheumatoid nodules. Particular cutaneous manifestations correlate with disease activity and thus have great diagnostic value. However, diversity of the clinical picture and resemblance to certain entities delay making an accurate diagnosis The aim of this review is to discuss the variety of cutaneous manifestations and indicate the clinical features of particular CLE types which facilitate differential diagnosis with other dermatoses. Although in diagnostically difficult cases histopathological examination plays a key role in the differential diagnosis of LE, quick and accurate diagnosis ensures adequate therapy implementation and high quality of life for patients. Cooperation between physicians of various specialties is therefore crucial in the management of patients with uncommon and photosensitive skin lesions.

## 1. Introduction

Lupus erythematosus (LE) belongs to the group of autoimmune diseases with a chronic course including periods of exacerbations and remissions [1]. Skin manifestations are the second most common symptom of systemic LE (SLE) [2]. The current 2019 EULAR/ACR criteria for SLE list the mucocutaneous domain, one of seven clinical domains, including non-scarring alopecia, oral ulcers, subacute cutaneous or discoid lupus and acute cutaneous lupus [3], which are attributed a total of 14 points. An initial diagnosis of cutaneous LE (CLE) is associated with a 5–18% risk of developing SLE over the next 3–5 years, and these patients usually present with a mild course of the disease [4,5,6,7]. Concurrently, CLE may occur as a separate entity in the absence of systemic symptoms, and such a variant is characterized by a 2–3-fold higher incidence compared to SLE [8,9]. Genetic and environmental factors play a key role in the pathogenesis of CLE, as in SLE [10].

Gilliam and Sontheimer divided CLE based on the clinical picture into three types: acute CLE (ACLE), subacute CLE (SCLE) and chronic CLE (CCLE), which constitute the specific lesions [11,12]. Non-specific lesions, such as Raynaud’s phenomenon, periungual teleangiectasias, livedo reticularis or leukocytoclastic vasculitis, are also frequently observed in CLE [13]. Diagnosing CLE requires proper classification of skin lesions into one of the three subtypes mentioned above. For now, there are no standardized diagnostic criteria for each subtype of CLE. The key diagnostic tool is histopathological examination of a skin specimen supported by typical features of the clinical picture and serologic abnormalities [13]. The typical histopathological pattern found in CLE is vacuolar interface dermatitis involving the dermo-epidermal junction, with a few exceptions [14]. Furthermore, the medical history of the patient should also be comprehensively assessed. In the case of CLE, direct immunofluorescence (DIF) is somewhat less important. Prior to making a diagnosis, possible involvement of internal organs should be assessed, as specific variants of CLE, i.e., malar rash, may be part of the broad spectrum of the clinical picture in SLE [15].

The aim of this review is to present the cutaneous manifestations of LE, which can range from mild, self-limited to severe lesions and to draw the attention of physicians of various specialties mainly to less-common and less-known forms of cutaneous lesions.

## 2. Acute Cutaneous Lupus Erythematosus

The most frequent and commonly known entity in the ACLE group is malar rash, which is characterized by notable photosensitivity. There are also other less-specific variants [14]. Histopathologically, vacuolar interface dermatitis, mild lymphocytic infiltrate and dermal edema are typical of ACLE. Serologic testing may reveal the presence of antibodies directed against double-stranded DNA (anti-dsDNA), Smith antigen (anti-Smith), U1 ribonucleoprotein (anti-U1RNP) and/or anti-Ro antibodies. In the vast majority of cases, the lupus band test, which is a DIF technique, is positive. ACLE shows a strong association with SLE [14]. Anti-dsDNA antibodies in combination with low levels of complement proteins, particularly C3 and C4, are associated with disease activity and can be used as markers for lupus flares [16]. Skin lesions in SLE are directly caused by immunoglobulin deposits. The predominant types of immune complexes consist of IgM alone or a combination of IgM and C3. Particularly noteworthy is that IgM is the most prevalent immunoglobulin identified in skin lesions of SLE patients [17].

### 2.1. Malar Rash

Malar rash, also referred to as butterfly erythema, is a localized form of ACLE. It occurs primarily in women in the third decade of life and is frequently associated with active SLE [18,19]. It presents as symmetrical erythematous and edematous lesions on the cheeks, bridge of the nose and, less frequently, forehead and front of the neck. The nasolabial folds are spared [9,20]. Lesions are transient, non-scarring and usually induced by ultraviolet (UV) radiation. Rarely, malar rash may be accompanied by post-inflammatory dyspigmentation, especially in the dark-skinned population [13,21]. 

Histopathological findings include superficial perivascular lymphocytic infiltration and basal vacuolization [22]. Malar rash should be differentiated from sunburn, rosacea, seborrheic dermatitis, acne and eczematous dermatitis [2]. However, in rosacea, papules and pustules are observed, while in seborrheic dermatitis, the nasolabial folds are affected [13]. Sunburn manifests as edematous and purpuric lesions. It also may induce painful blister formation and skin necrosis with subsequent defects in the epidermis and dermis, as well as scars. The primary lesion in acne vulgaris is a comedone, which evolves into inflammatory papules, pustules and nodules [23]. In eczema, in addition to typical lesions, vesiculo-bullous eruptions may also be observed. These features facilitate the differentiation of CLE from morphologically similar disorders [24].

### 2.2. Bullous Lupus Erythematosus

Bullous lupus erythematosus (BLE) is a subepidermal bullous disease characterized by the presence of antibodies directed against collagen VII. It mainly affects young women, which accounts for 5% of SLE patients [18]. An acute onset of lesions is typical of BLE [25]. Extensive and tense vesiculo-bullous lesions develop, particularly affecting areas exposed to UV radiation, i.e., face, neck, upper trunk, upper limbs, red zone of the lips and mucous membrane of the oral cavity [18,25]. However, bullous lesions may also appear on the skin of other parts of the body [9]. Bullae are located on erythematous-background or normal-looking skin and resolve without scarring or milia formation. Nevertheless, layered crusts remain in the blister sites of the skin. Common cutaneous manifestations often coexist with renal involvement and severe course of the disease [18]. In a number of cases, glomerulonephritis has been diagnosed simultaneously or after the BLE, suggesting that vesico-bullous lesions are a sign of disease flare [26,27,28]. Generally, pruritus does not occur. Unlike other forms of LE that may proceed with blister formation, such as the Stevens–Johnson syndrome/toxic epidermal necrolysis (SJS/TEN) variant of lupus described below, BLE responds rapidly to dapsone. The differential diagnosis includes dermatitis herpetiformis, bullous pemphigoid and linear IgA bullous pemphigoid [9]. In addition to the typical histopathological features in CLE, BLE is also characterized by the presence of subepidermal bulla with neutrophils in the blister cavity and dermal papillae. DIF examination may show linear or granular immune deposits along the basement membrane [14].

### 2.3. Stevens–Johnson Syndrome/Toxic Epidermal Necrolysis Variant of Cutaneous Lupus Erythematosus

**Figure 1:**
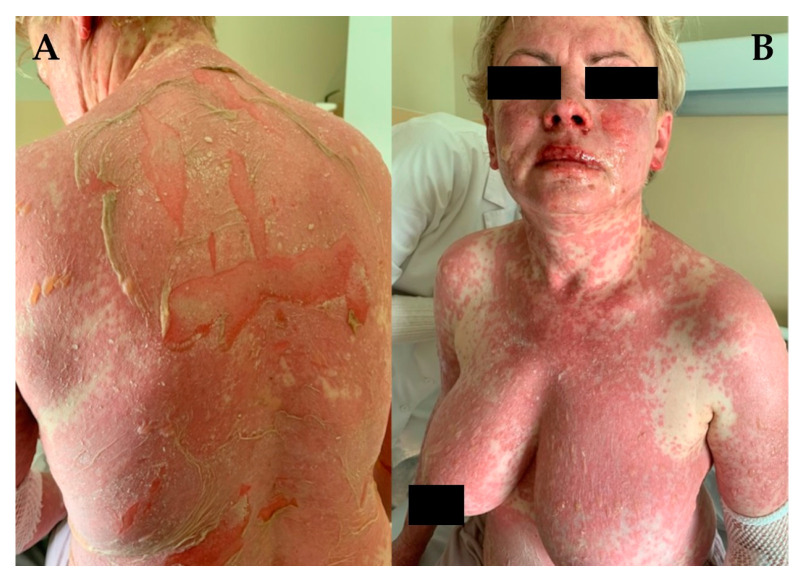
Toxic epidermal necrolysis, a variant of SLE maculopapular rash on erythematous skin with positive Nikolsky’s sign. (**A**,**B**) Typical large area of skin involvement.

The SJS/TEN variant of CLE is a subtype of ACLE as well as SCLE, usually when a severe course of the disease exists. It appears in both CLE and SLE [9,29]. The clinical presentation includes macules, papules and erythematous lesions affecting large areas of the body (Figure 1), within which blisters may form [18]. Furthermore, erythematous-scaling plaques with distinct photodistribution may occur. Some of the lesions show central clearing [30,31]. Contrary to the classic drug-induced form of SJS and TEN, drug unrelated skin lesions in the SJS/TEN variant of CLE occur as a result of intense sun exposure [31]. It develops on the basis of previously diagnosed ACLE and SCLE or undetermined autoimmune disorders. The skin lesions evolve gradually from typical LE lesions or develop de novo, which is a more rapid process [32,33]. Skin involvement resolves without scarring but sometimes leaves post-inflammatory dyspigmentation [18]. Due to the positive Nikolsky’s sign, although not always present in this variant, the disease should be differentiated from classic SJS and TEN [9,34]. In contrast to these conditions, the SJS/TEN variant of CLE presents with uninvolvement or slight involvement of the mucous membranes, no clear correlation with medication intake, as previously mentioned, and a better prognosis [29]. Differential diagnosis is usually supported by histopathological examination, which typically shows interface dermatitis with keratinocytic cell death, which is a differentiating factor with other ACLE subtypes, as well superficial perivascular and periadnexal lymphocytic infiltrate with interstitial mucin deposition [14,30,31].

### 2.4. Maculopapular Rash

**Figure 2:**
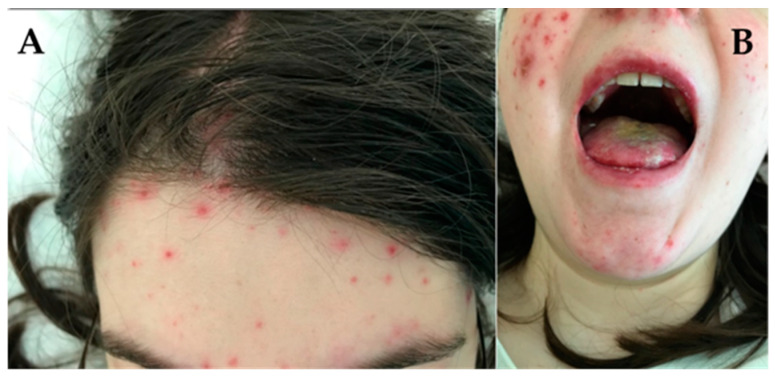
Acute cutaneous lupus erythematosus. (**A**) Erosions, macules and papules partially covered with scab on an erythematous base located on forehead, cheeks and chin resembling a drug or viral eruption. (**B**) Erosions on the oral mucosa.

This variant represents a generalized form of ACLE. The pruritic rash is symmetrically distributed on the skin above and below the neck [9]. Usually the extremities, chest and shoulders are involved. Additionally, hypertrophic cuticle, erythema and dilated or atrophic vessels in the periungual location are frequently reported [13,18]. The morphology and location of lesions are similar to those in drug-induced (Figure 2) photosensitivity or measles [18].

### 2.5. Erythema after Exposure to Ultraviolet Radiation

Erythema appears in patients with hypersensitivity to UV radiation. The photodistribution of skin lesions is typical. The intensity of erythematous lesions shows no correlation with the duration of exposure to sunlight. Moreover, erythema occurs over various periods of time after photoprovocation, from several days to even several weeks. Therefore, the causal connection between the exposure to sunlight and induction of skin lesions often remains undetected [18]. The presented form of ACLE, similarly to dermatomyositis (DM), may affect the dorsal surface of the hands. When differentiating these entities, it should be noted that in DM the Gottron sign is observed on the skin over the interphalangeal joints, while in ACLE these sites are spared from skin lesions [19,20].

## 3. Subacute Cutaneous Lupus Erythematosus

The incidence of SCLE is relatively high compared to other variants of LE. As a general rule, its classic forms do not pose diagnostic difficulties in everyday medical practice. SCLE, as SLE, is diagnosed mainly in the female population of reproductive age [13]. In approximately 50% of SCLE cases, patients also meet the criteria for SLE. However, they present with a mild course of the systemic disease, mostly including arthritis and myalgias [35,36]. SCLE shows a strong correlation with the presence of antinuclear antibodies (ANA), especially anti-Ro/SS-A, and more than 50% of SCLE patients have coexisting Sjögren’s syndrome [19,37,38,39]. Concurrently, Verdelli et al. in their study showed a strong correlation between SCLE and the presence of anti-SM and anti-RNP [40]. Histopathology in SCLE reveals vacuolar interface dermatitis with many cytoid bodies and also more prominent and deeper perivascular and perieccrine infiltration of inflammatory cells compared with ACLE [22]. However, in SCLE the presence of dermal lymphocyte infiltrate is lower compared to DLE, and periadnexal infiltrates are nearly absent [41]. In DIF, a positive lupus band test is observed in 65–80% of cases, and sometimes dust-like particles occur [14]. Deposits of IgM, IgG and C3 may be present. Despite the fact that 40–50% of patients with SCLE meet ACR criteria for SLE, only 10–15% of them will in fact develop the systemic form of the disease [14].

Common photosensitivity among patients with SCLE results in lesions located in sun-exposed areas, such as the upper trunk and extensor surfaces of the upper limbs. The midface, scalp and areas below the waist are usually spared [2]. There are few reports of lesions occurring in the oral cavity [42]. Two variants of SCLE are distinguished: papulosquamous and annular [25]. 

**Figure 3:**
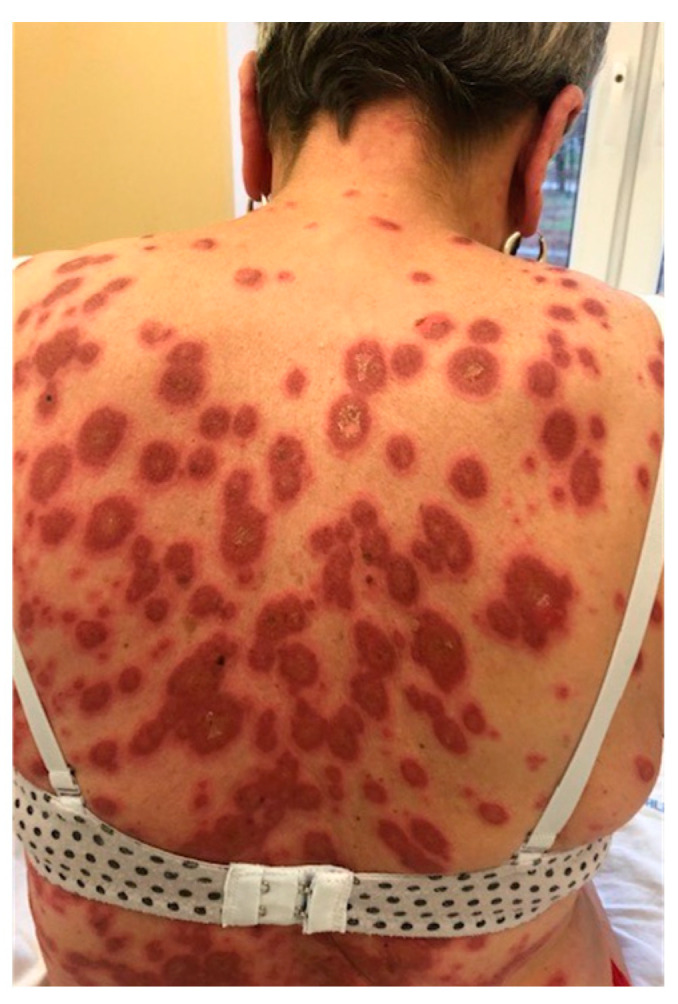
Subacute cutaneous lupus erythematosus, papulosquamous variant. Erythematous plaques with superficial scaling.

**Figure 4:**
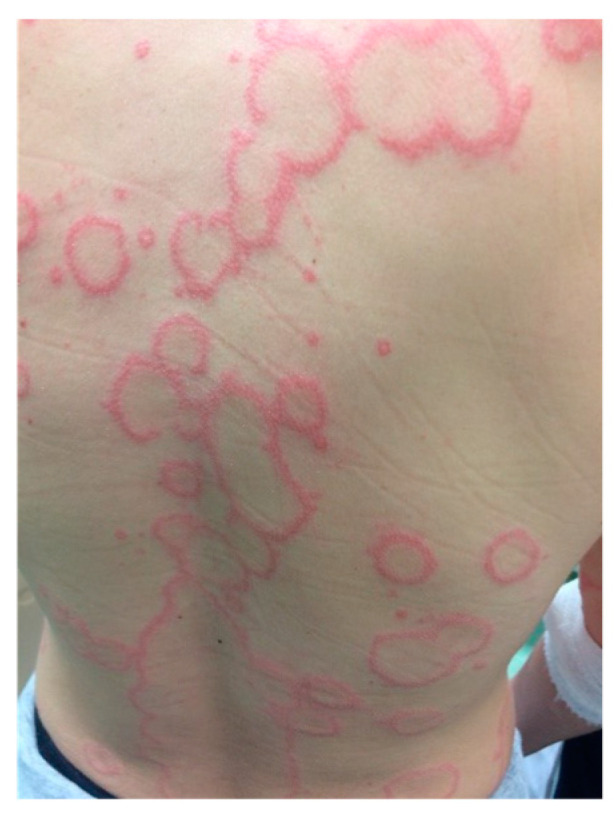
Subacute cutaneous lupus erythematosus, annular variant. Annular lesions with rims of erythema.

The former is characterized by erythematous-papular lesions with superficial scaling, which may evolve into psoriasis-like plaques [25] (Figure 3). Papulosquamous SCLE may be mistaken for eczema, psoriasis and pityriasis [13]. In the latter, erythematous annular plaques with a tendency to coalesce, accompanied by central brightening and peripheral scaling, are detected [25] (Figure 4). Typical lesions in psoriasis are annular plaques on erythematous skin with superficial silver scaling. In nummular eczema, reddish, scaly, coin-shaped lesions occur, and in pityriasis rosacea, annular or ovoid lesions with peripheral scaling occur. Concurrently, annular lesions in SCLE may be atrophic with slight scaling [43]. Despite inconsistencies in the results of various studies, it is suggested that the annular variant is more common [44,45,46,47]. Nevertheless, both types of lesions may coexist. Such a condition may be associated with the appearance of peripherally distributed vesicles, crusts and hemorrhagic blisters and is referred to as “lupus with bullae” [19,46]. The lesions in SCLE persist even for several months and heal without scarring or skin atrophy, but they may cause postinflammatory hypopigmentation, clinically resembling vitiligo [48].

SCLE should also be differentiated from DM. In DM, typical characteristics are Gottron’s sign and periorbital erythema, which are not classic manifestations of CLE. Concurrently, common features of the clinical picture in DM are facial erythema and V-neck erythema. In most cases, histopathological pictures of skin specimens taken from patients with DM and CLE are indistinguishable [49,50]. However, with special immunohistochemical or transcriptomics techniques, it is possible to reveal the following histopathological features: myeloid dendritic cell infiltration in DM and plasmacytoid dendritic cell infiltration in CLE. Nevertheless, these are laboratory methods with very limited availability, making them not commonly used in many laboratories. For this reason, histopathological examination is not the best tool in the differential diagnosis of DM and SCLE [51]. 

It is worth emphasizing that there is a subtype of drug-induced SCLE (DI-SCLE). Differentiation from idiopathic SCLE may be problematic because the clinical presentation is identical in both variants. Therefore, a detailed medical and drug history of the patient should be taken. The key triggering factors are medications that were started within a few weeks to 9 months from the onset of skin lesions. The literature shows that antihypertensive drugs, as hydrochlorothiazide, angiotensin-converting enzyme inhibitors, calcium channel blockers, the antifungal agent terbinafine and also to a lesser extent tumor necrosis factor-alpha antagonists, immunosuppressants and chemotherapeutics remain crucial in the pathogenesis of DI-SCLE [52,53].

Skin lesions in DI-SCLE affect wider areas of the body than in non-drug-induced SCLE. The differential diagnosis between these two variants is of great significance due to management discrepancies. In DI-SCLE, the exclusion of the suspected drug is usually adequate, while idiopathic SCLE requires systemic treatment [54]. Other entities that can resemble SCLE are cutaneous T-cell lymphoma, tinea, erythema annulare centrifugum, erythema gyratum repens, photolichenoid drug eruption, granuloma annulare and pemphigus foliaceus [13]. 

Rare and little-known forms of SCLE include:

### 3.1. Pityriasiform SCLE

Clinically pityriasiform SCLE manifests as erythematous and squamous lesions on areas exposed to UV radiation. Caproni et al. described a case of a 57-year-old woman who presented with numerous small erythematous patches coalescing into one large lesion with irregular borders on the upper back. There were a few small areas of unaffected skin within it. Superficial scaling and varying infiltration were observed in the reddish lesions. In addition, single, erythematous, reticular-like lesions with distinct pityriasiform scales were also detected on the skin of the trunk. In laboratory tests, positive anti-dsDNA (titer 1: 160) and anti-Ro/SSA antibodies were detected [55]. 

Similarly to acute LE, the patient reported sun-induced facial erythema sparing the nasolabial folds accompanied with slightly scaling surface. Another symptom found was telangiectasia on the skin of the face, trunk, limbs and periungual area. Histopathological examination revealed epidermal atrophy with hyperorthokeratosis, hydropic degeneration of the basal cell layer, perivascular lymphocytic infiltration and cellular edema of the upper layer of the dermis [55].

### 3.2. SCLE Resembling Vitiligo

Pigmentation abnormalities induced by abnormal T lymphocyte activity typically occur in vitiligo. The same immune cells are involved in the pathogenesis of LE. Niebel et al. described a case of a 59-year-old woman with SCLE diagnosis confirmed by skin biopsy. The patient was treated with hydroxychloroquine. As a result, quick resolution of the scaling erythema, located on the upper back and extremities, was observed. However, depigmentation of the initially affected skin appeared within two months with following slight repigmentation spreading centripetally. Antimalarial drugs, the standard treatment for LE, have a high affinity for melanin and, therefore, impair melanogenesis [56].

### 3.3. SCLE as Generalized Poikiloderma

Poikilodermatous SCLE usually develops on originally unaffected skin after sunburn. Poikiloderma appears as reticulated brownish areas of hypopigmentation and hyperpigmentation, telangiectasia, atrophic changes, slight wrinkling and superficial scaling. Other observed symptoms may include psoriasiform or miliary lichenoid papules and small petechial hemorrhages. Poikilodermatous lesions are localized on sun-exposed areas, such as the face, scalp, trunk and extensor surfaces of extremities [57,58].

### 3.4. Exfoliative Erythroderma-like SCLE

Erythrodermic SCLE is characterized by erythematous plaques that evolve from psoriasiform lesions, most often after exposure to sunlight. Physical examination usually detects intense scaling. Other observable elements of the clinical picture are annual or polycyclic SCLE lesions. It is generally accepted that erythroderma involves more than or equal to 90% of the body surface area [59,60]. Pai et al. report a case of a 58-year-old male patient with exfoliative erythroderma-like SCLE, presenting with extensive scaling, mainly involving the scalp, face, chest and back. The scaling was accompanied by ecthymatous ulcers on the skin of his back, abdomen and extremities [61].

## 4. Chronic Cutaneous Lupus Erythematosus

CCLE most frequently presents as discoid lupus erythematosus (DLE) with typical cutaneous manifestations. There are also many other variants of CCLE with highly low incidence and distinct symptoms. Immunological processes underlie the pathogenesis of these skin lesions. Studies have indicated that IgG is the predominant immune complex found in DLE [62]. Additionally, compared to SLE, DLE patients tend to have fewer immune complexes. These findings collectively suggest a lesser involvement of IgM in the skin damage of DLE compared to SLE [63].

### 4.1. Discoid Lupus Erythematosus

Discoid lupus erythematosus (DLE) is the most frequent type of chronic CLE, and it is estimated that it may constitute from 50 to 85% cases of CCLE [64]. It can be divided into two forms: localized (DLE) and disseminated (DDLE). In its localized form, skin lesions are located on the scalp, face, external canal and conchal bowl of the ears, anterior neck and extensor arms, whereas in its disseminated form skin lesions are located above and below the neckline, and typical predilection sites are the extensor forearms and hands [20]. 

**Figure 5:**
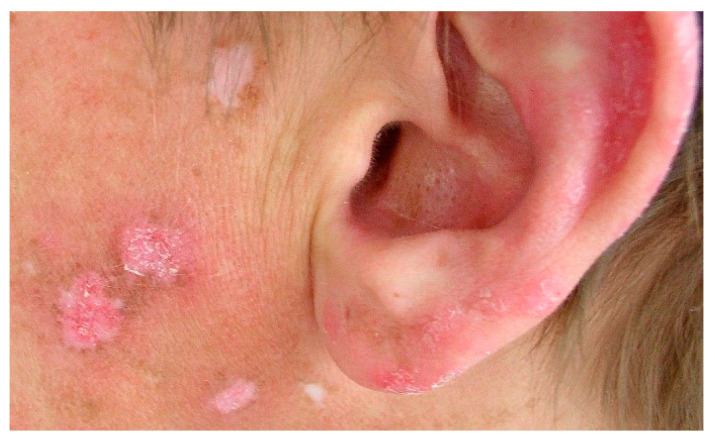
Chronic cutaneous lupus erythematosus. Discoid lupus erythematosus. Erythematous lesions with follicular hyperkeratinization and peripheral hyperpigmentation.

Initially, there is a purplish macule or papule, which transforms into a discoid or coin-shaped plaque with peripheral hyperpigmentation [19] (Figure 5). In this condition, the hair follicle can be clogged by adherent scales, and peeling the scale reveals keratotic spikes, which is called ”carpet tack sign”. The progression of this phenomenon destroys hair follicles permanently and may result in scarring alopecia. Over time, skin lesions become atrophic with peripheral discoloration and central depigmentation and cause irreversible and severe skin defects [65].

Triggers exacerbating DLE include UV radiation, cold, injuries, infections and burns. DLE affects most often people between 20 and 40 years old, twice as many woman than men [66]. It is estimated that from 17 to 30% patients with DLE can develop SLE in the future, and approximately 8–28% patients with SLE have discoid lesions [12,66,67]. However, patients with localized DLE have a lower risk of developing SLE than those with DDLE [66]. Also, periungual lesions in DLE predispose to SLE [39].

DLE at an early stage should be differentiated from psoriasis, lymphocytoma cutis, cutaneous T-cell lymphoma, granuloma faciale, polymorphous light eruption and sarcoidosis [13]. In psoriasis, skin lesions are round, covered with plaques and are distributed in areas exposed to injuries [68]. Granuloma faciale is a brown-red plaque located on the face and has a smooth surface with follicular orifices and telangiectasia [69]. T-cell lymphoma in contrast to DLE appears in non-sun-exposed areas. Lymphoma cutis in turn has a wide range of clinical pictures from macule to nodules, papules, patches and plaques, induration or erythroderma [70]. 

If the clinical features are uncertain, DIF and histopathology of affected skin should be performed. DIF reveals deposits of complement or immunoglobulins at the dermal–epidermal junction in 50–90% of cases [71]. Histopathological findings contain vacuolar to lichenoid interface dermatitis with adnexal involvement, follicular plugging and hyperkeratosis. The basement membrane is thickened, and dermal mucin is increased [72]. Moreover, serology shows the absence of ANA or ANA in low titer [14].

Other entities that should also be considered in the differential diagnosis are actinic keratosis (AK), basal cell carcinoma (BCC), squamous cell carcinoma (SCC) and infections, such as leishmaniasis or deep fungal infections. In AK, unlike DLE, histopathology can show focal parakeratosis, loss of the granular layer, dilated follicular openings, dysplastic keratinocytes, focal acantholysis and moderate elastosis [73]. Moreover, a “strawberry” pattern is observed in dermoscopic images of both AK and SCC, which consists of a reddish background intermingled with white, enlarged follicular openings [73,74]. In DLE there is rather a pink background interrupted by reddish, follicular openings, surrounded by a white halo [73]. BCC is differentiated from DLE based on typical histopathological features, such as mucin deposition around the tumor and peripheral palisading of cells with central horn pearl. Lesions in leishmaniasis, such as papules and ulcers, tend to resolve spontaneously, which is typically not seen in DLE [75]. Fungal infections are characterized by eosinophilic infiltrates in histopathology, while in DLE, the absence or rare presence of eosinophils is typical [76]. 

An important factor differentiating CCLE from other skin conditions is the presence of plasmacytoid dendritic cells (PDC) with surface receptor, the alpha chain of the interleukin-3 receptor (CD123). The involvement of CD123+ PDCs in the pathogenesis of LE is supported by their ability for intensive production of type I interferons. Importantly, the active phase of CLE is characterized by hyperactivation of the type I interferon pathway, which enhances the immune response targeting the affected skin. Unlike inflammatory dermatoses and neoplasms affecting skin, PDC infiltration is much larger in CLE and consists mainly of single cells. While in other entities, PDC are usually grouped into clusters. Moreover, CLE is characterized by perivascular, periadnexal and dermo-epidermal distribution of PDC [77,78,79].

Rare forms of CCLE include those listed below.

### 4.2. Hypertrophic/Verrucous Discoid Lupus Erythematosus

Hypertrophic/verrucous discoid lupus erythematosus is a rare variant of DLE. Hypertrophic and verrucous lesions occur in only 2% of patients with CLE [80]. It manifests as pruritic, erythematous and strongly indurated plaques with scaling and infiltration [2,80]. Skin lesions are located mainly on the extensor surfaces of the upper limbs and less frequently on the face and upper trunk [81]. Hypertrophic (verrucous) lesions usually develop on the basis of long-term and untreated DLE eruptions and are associated with an increased risk of skin cancer [18].

Presented variants of DLE can resemble keratoacanthoma, hypertrophic LP, SCC and pseudoepitheliomatous hyperplasia (PEH) associated with other diseases [13]. Therefore, differential diagnosis is very important, and histopathological examination is often necessary. Histopathology reveals follicular lesions with hyperkeratosis and periapical lymphocytic infiltration. Irregular epidermal proliferation almost always occurs as well as vacuolar degeneration of the basement membrane zone. Compared to other CLE subtypes, localized amyloid deposits are more frequently observed [82]. Like in DLE, the lupus band test is positive in 50–90% of cases, and ANA are also absent or present in low titers [14].

### 4.3. Lupus Erythematosus Panniculitis

**Figure 6:**
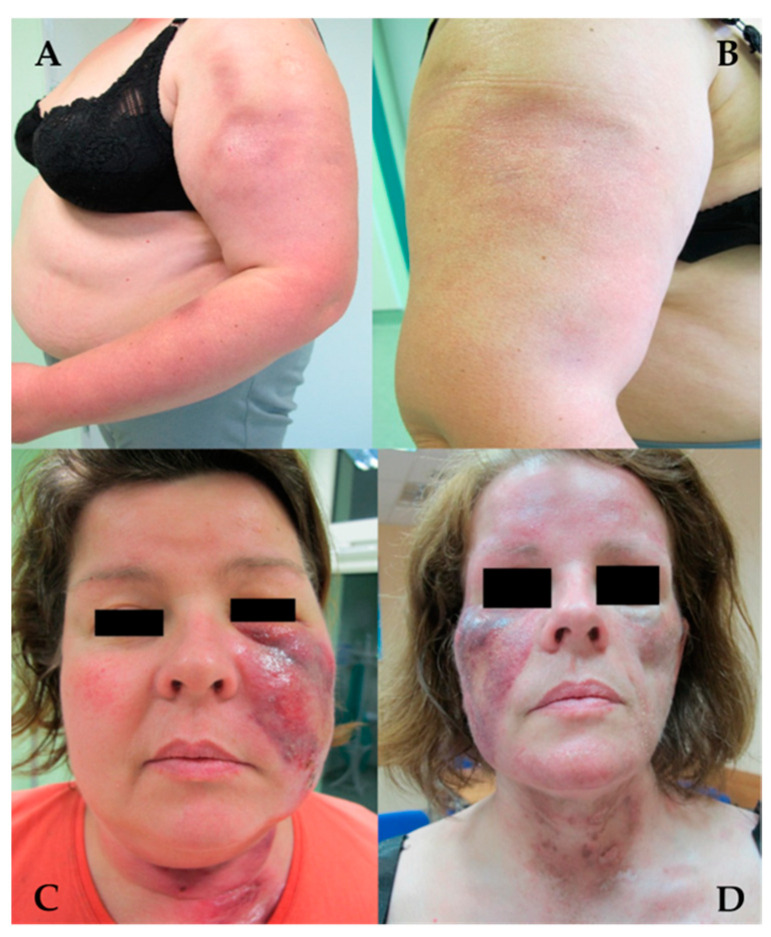
Chronic cutaneous lupus erythematosus. Lupus panniculitis. (**A**,**C**) Nodular infiltration with edema and erythema. (**B**,**D**) Deep atrophic areas after resolution of skin lesions.

**Figure 7:**
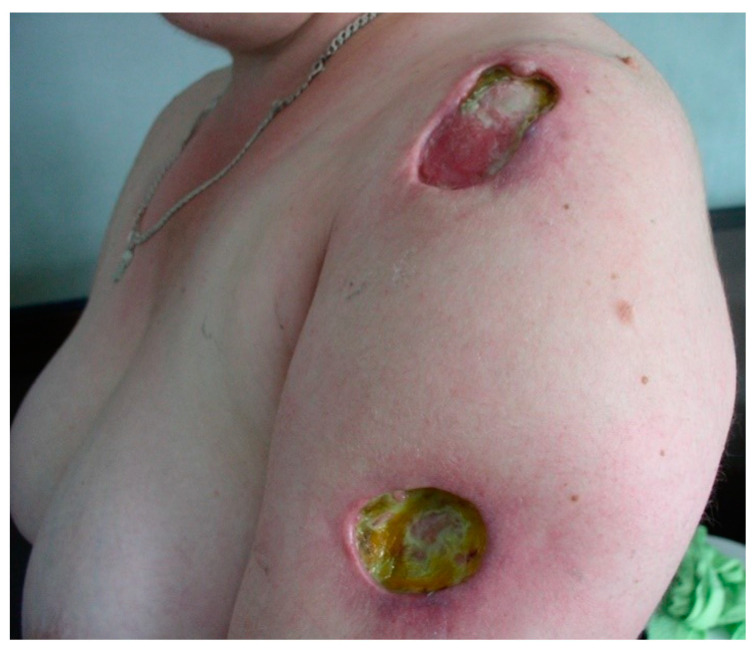
Chronic cutaneous lupus erythematosus. Lupus panniculitis. Deep ulceration with a granulating base, partially covered by fibrin.

LEP may occur as a separate disease or overlap with DLE or SLE [83]. Clinically, it is characterized by painful, indurated subcutaneous nodules occurring in areas of increased fat deposition, such as the arms and legs (gluteal region and thighs) and face [12,20,84] (Figure 6). Deeply located plaques are hard to the touch, and within time nodules or tumors lead to secondary development of atrophic scarring. Skin lesions may rarely take the form of calcifications and ulcerations [85] (Figure 7). The clinical symptoms may also include erythema, poikiloderma or follicular hyperkeratosis [86]. 

The onset of the disease may be caused by various factors, mainly by physical injuries [86]. The course of the disease is chronic with exacerbations and remissions, and resolution of lesions leaves lipoatrophic areas. Lipoatrophic areas in characteristic locations, such as the shoulders and upper arms, allow for making a diagnosis without additional tests [87]. 

Upon histopathological examination, typical features include infiltration of lymphocytes, hyaline necrosis of the fat lobule and nuclear dust in infiltrating lymphocytes lobules of adipose tissue [88]. In cases of typical clinical picture, but non-diagnostic histopathological picture, the lupus band test (LBT) is recommended and deposits of immunoglobulins or C3 at the dermo-epidermal junction, detected in 70–90% of cases, support the diagnosis [89]. Another feature in favor of LEP is low ANA titers [14].

In contrast to other forms of CLE, no hypersensitivity to ultraviolet radiation is observed in patients [90]. This subtype must be differentiated from panniculitis-like subcutaneous T-cell lymphoma and subcutaneous sarcoidosis [85]. A relatively common variant of LEP is lupus mastitis (LM), which refers to the involvement of the breast adipose tissue. Another frequent localization of lesions in LEP is the scalp, which can be seen in sclerodermic linear LEP [91,92]. 

Clinically, LM presents as a subcutaneous nodules, which may develop into ulcers over time, resolving with atrophic scarring [93]. It is a rare manifestation and can present with subcutaneous lesions or deep masses that may mimic malignancy in a clinical picture and also in radiological findings because both mammography and ultrasonography show ill-defined density and coarse calcifications [94]. Therefore, the biopsy of skin should be performed, and lobular panniculitis and hyaline fat necrosis typically occur [93]. 

Sclerodermic linear LEP is another rare variant of LEP, and it is within the clinicopathological spectrum of linear morphea and cutaneous lupus erythematosus. Few distinguishing features have been proposed for this condition: linear distribution, young age of onset, Blaschko-linear assessment and a low risk of progression to systemic disease [95]. 

### 4.4. Chilblain Lupus 

**Figure 8:**
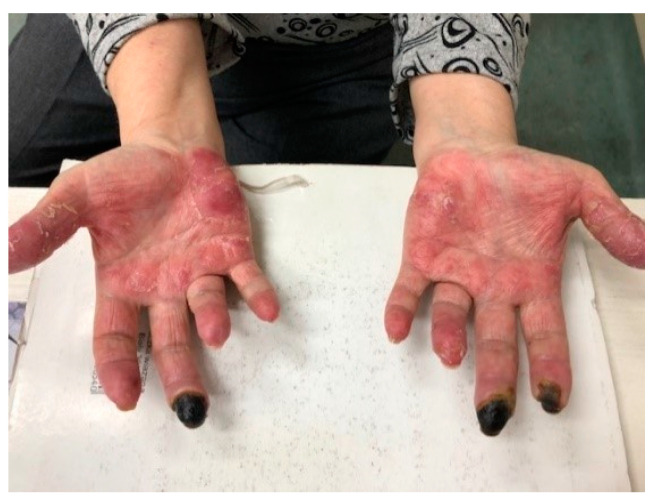
Chronic cutaneous lupus erythematosus. Chilblain lupus. Erythematous swelling and infiltrative eruptions, with difficult-to-heal erosions and ulcers on distal parts of the body.

CHLE is a rare form of CLE resembling a common chilblain. Skin lesions are purple-red or erythematous typically located on acral parts of the body: fingers, toes, heels, nose and ears [96]. Clinically, central erosions, ulcerations, painful plaques and nodules can also appear [20] (Figure 8). These lesions can develop after 12 to 24 h after exposure to trigger factor and are accompanied by burn and pain [97]. When palms and soles are involved, necrosis or fissures can be present [98].

The Mayo Clinic diagnostic criteria are used to confirm the diagnosis and presence of two from three major criteria (erythematous skin lesions caused by exposure to cold, acral location, presence of skin lesions characteristic to lupus erythematosus), and at least two from three minor criteria (coexistence with SLE or CLE, improvement after therapy, absence of cryoglobulins in the serum) must be met [99]. 

Systemic lupus erythematosus (SLE) may be diagnosed in 20% of patients with CHLE. This type of CCLE should be differentiated from acrocyanosis, primary Raynaud’s phenomenon, lupus pernio Besnier, livedo reticularis, DM, equestrian cold panniculitis, cold urticaria, perniosis (usual chilblains), idiopathic chilblains, COVID toes and also lesions related with interferonopathies, as familial chilblains, Aicardi–Goutières syndrome and STING-associated vasculopathy [100,101,102,103,104,105,106,107,108,109,110,111,112]. Specific histopathological features include papillary edema, angiocentric and reticular dermal lymphohistiocytic infiltrate, as well as features reminiscent of lupus, such as interface dermatitis and degeneration of the dermo-epidermal junction with deposition of immunoglobulins (IgM, IgA) and complement (C3). Additionally, there are perivascular deposits of C3 and fibrinogen. However, histopathological findings in CHLE might be very problematic to differentiate from a common chilblain or COVID toes [113]. While the results of the lupus band test are variable, the serological test mostly shows positive anti-dsDNA and anti-Ro antibodies [14].

### 4.5. Lupus Tumidus 

LET is a subtype of CLE which is related to extreme photosensitivity. Elevated, infiltrated and edematous plaques with smooth surface appear on the skin, especially in sun-exposed areas [48]. Plaques have central clearing and raised borders and are described as “urticarial plaques” [9]. The clinical course consists of alternating relapses and remissions in relation to ultraviolet radiation. The lesions resolve without any scars because there are no epidermal changes [48].

LET rarely co-occurs with SLE, but it often coexists with other subtypes of CLE. Some authors have proposed a separate category for LET—intermittent cutaneous lupus erythematosus (ICLE)—because of significant differences between clinical course, antinuclear antibodies ratio and histopathological findings in LET and other subtypes of CLE [114]. 

However, according to criteria from Systemic Lupus International Collaboration Clinics (SLICC), LET is classified as one of the forms of chronic CLE [115]. Clinically, this form of lupus can mimic reticular erythematous mucinosis (REM), polymorphic light eruption (PLE), lymphocytic infiltration of the skin Jessner–Kanof (LIS) and cutaneous lymphoproliferative disorders. Histopathological examination shows superficial and deep perivascular and periadnexal lymphocytic infiltrates with prominent mucinous dispositions. There are no or minimal epidermal changes [116]. The lupus band test results are variable, and ANA is often absent [14].

### 4.6. Discoid Lupus/Lichen Planus Overlap

The DLE/LP overlap syndrome is a rare disorder that combines the morphological and histopathological features of both diseases [117,118]. It is most frequently diagnosed in the age group 25–45 with a slight female predilection [119]. Two clinical forms are distinguished. The first variant is characterized by painful, itchy, scaly blue-reddish plaques and macules with central depigmentation detected on the extremities. The second variant presents as papulo-nodular lesions located on the upper limbs [120]. Skin lesions in DLE/LP overlap syndrome may also develop on the face, scalp and nail plates [9]. Histological findings include features of LP (hyperkeratosis, hypergranulosis, irregular acanthosis, vacuolar degeneration of the basal cell layer, pigment incontinence) with or without features of CLE (hyperkeratosis, vacuolar degeneration of the basal cell layer, follicular plugging, lymphoid infiltrate, interstitial mucin). Identifying this subtype is often difficult because there are not clear diagnostic criteria [118]. 

### 4.7. Comedonic Lupus Erythematosus

Comedonic lupus erythematosus is a very rare variant of CLE. Only a few cases of comedonic LE have been described in the literature, mostly in women in the third decade of life [121]. Skin lesions are described as comedones, erythematous, pruritic or acneiform papules and pustules located in seborrhoic and sun-exposed areas (face, ears or chest). Skin lesions are often accompanied by pruritus [122]. This condition is often mistakenly recognized as acne vulgaris and treated without clinical response [123]. Therefore, CCLE should be considered when acne lesions appear, which are itchy and do not improve after standard treatment. Histopathological examination may help in differential diagnosis, which reveals vacuolar degeneration of the basal layer, perivascular and periadnexal lymphocytic infiltrates and also acanthosis and atrophy of the epidermis [123]. 

### 4.8. Acneiform Lupus Erythematosus

Acneiform lupus erythematosus is one of the most scarcely observed forms of CLE. Based on the case report of a 32-year-old woman reported by Vieira et al., it was concluded that CCLE can manifest with infiltrative acneiform and comedonal lesions accompanied by pruritus. Physical examination revealed the presence of erythematous-infiltrative lesions, papules and open comedones, which are the primary lesion in acne vulgaris. Pitting scars and hypopigmented atrophic scars were also detected. The skin lesions were located mainly on the right chin. The face is one of the most common areas affected in both acne vulgaris and CCLE. One of the most important features distinguishing the acneiform variant of CCLE from acne vulgaris is the lack of therapeutic effect after standard anti-acne treatment with isotretinoin and the presence of pruritus [123]. In another case report of a 19-year-old woman, Uzuncakmak et al. described cicatricial acneiform lesions bilaterally on the cheeks and subcutaneous nodular lesions located proximally on the upper and lower extremities. Laboratory tests revealed ANA in a titer of 1:320, including anti-dsDNA present. Considering the clinical picture, laboratory and histopathological findings, such as septal thickening and lobular inflammation rich in histiocytes, as well as periadnexal inflammation with focal granulomatous reaction, CLE was diagnosed [124]. 

### 4.9. Rosacea-like Lupus Erythematosus

There are very few case reports of rosacea-like variants of CLE in the available literature. However, each patient presented with erythema in the central part of the face and erythematous or erythematous-purplish papules, 2 to 3 mm in diameter, within the skin of the cheeks and forehead. Moreover, patients complained of an intense burning sensation and, less frequently, slight to moderate itching. The rosacea-like variant of CLE is characterized by sudden onset and exacerbation induced by exposure to sunlight. Treatment commonly used for rosacea, including tetracyclines, azithromycin or both oral and topical metronidazole, had been unsuccessful, unlike antimalarials, which induced resolution of skin lesions in all cases [125].

### 4.10. Mucosal Lupus

**Figure 9:**
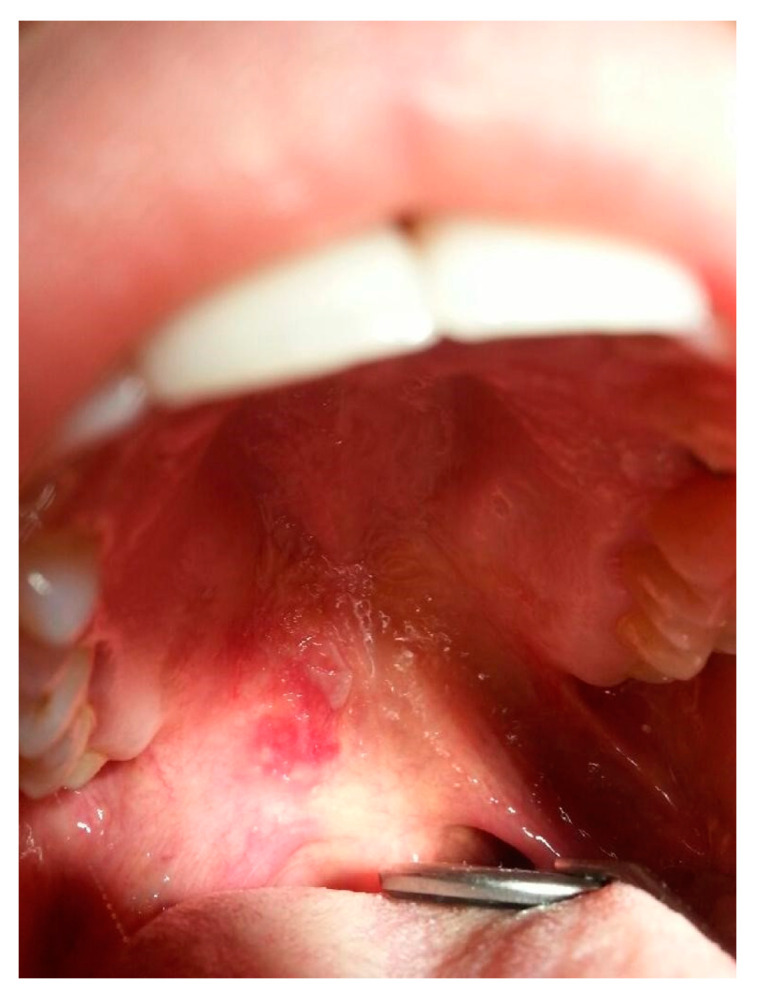
Mucosal SLE. Erosions on erythema lesions.

Oral mucosa involvement is found in 3–25% of CLE cases [126]. Mucosal lupus commonly presents as white papules forming a tree-like pattern, erosions and ulcers (Figure 9) affecting buccal and gingival mucosa or the red zone of the lips. Involvement of the genital mucosa is rarely reported. Lesions are usually observed in mechanically irritated sites. They are usually symptomless but seldom may cause mild hypersensitivity and dryness of the oral cavity [18,126]. Furthermore, the available literature includes numerous case reports of squamous cell carcinoma development within mucosal lupus lesions, especially in the red zone of the lips [127,128,129,130,131,132,133,134].

For instance, mucous membranes may be affected in DLE, and in cases of the buccal mucosa involvement, it may resemble lichen planus (LP) and oral leukoplakia, but the main distinguishing features are the radial arrangement and brush-like distribution with central erythema [84]. Genital involvement is rare [72]. In the differential diagnosis of the mucosal DLE histopathological criteria established by Lever are hyperkeratosis, atrophy of the Malpighian layer, hydropic degeneration of the basal cells, lymphoid cell infiltrate, features of edema and vasodilatation [135]. Another disorder in whose clinical picture mucosal lesions are observed is DLE/LP overlap syndrome [9].

## 5. Non-Specific Lesions

Non-specific lesions are not included in the diagnostic criteria of LE. Nevertheless, these are a common element of the clinical picture and provide additional confirmation of the diagnosis of LE. Non-specific skin lesions imply various complaints among patients and significantly reduce the quality of life [25]. Non-specific lesions usually correlate positively with an increased risk of internal organ involvement. Moreover, they may be a useful indicator of SLE activity [40].

### 5.1. Vascular Abnormalities

The most common vascular abnormalities are Raynaud’s phenomenon (a vasomotor disorder mainly affecting fingers during exposure to cold, including three stages: pallor, cyanosis and painful rubor), periungual telangiectasias (dilated, tortuous capillary loops), livedo reticularis (red-cyanotic macules forming a reticulate pattern on the skin), leukocytoclastic vasculitis (inflammation of small cutaneous vessels manifested as macules, papules and purpuric lesions) and urticarial vasculitis (urticarial lesions lasting longer than 24 h, which may evolve into purpura) [2,25]. Histopathology shows such abnormalities as angiocentric neutrophilic infiltrate with leucocytoclasis, fibrinoid necrosis of vessel walls and erythrocyte extravasation, whereas DIF reveals granular immune deposits in vessel walls. Cutaneous vasculitis occurs in nearly one third of patients with SLE [14].

### 5.2. Alopecia

**Figure 10:**
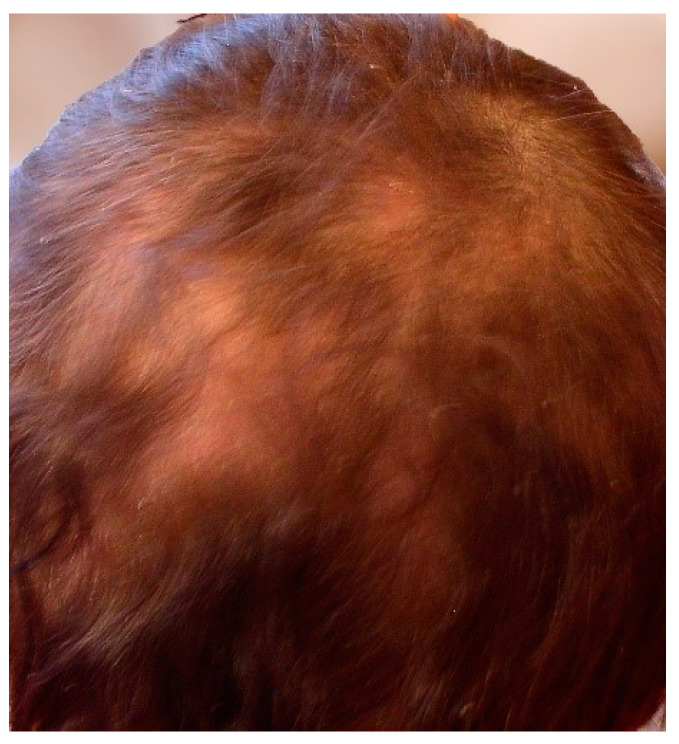
Non-scarring alopecia.

Focal hair loss is observed in telogen effluvium. Its severity is usually correlated with the activity of LE. In SLE exacerbations, excessive trichorrhexis, dryness and thinning of hair occur (Figure 10). This condition, which is dominant in the frontal hairline, is referred to as “lupus hair”. Trichoscopy may be crucial in differentiating from scarring alopecia resulting from focal lesions in the course of DLE. A dermoscopic image of “lupus hair” shows thinning and hypopigmentation of the hair shafts, telangiectasia and red dots around the hair follicles, white dots, honeycomb pigment macules and polymorphic interfollicular vessels [2,25,136].

### 5.3. Interstitial Granulomatous Dermatitis

LE-associated interstitial granulomatous dermatitis (IGD) is observed with very rare frequency. The clinical picture is notable for large plaques distributed symmetrically on the extremities and trunk. In addition, subcutaneous linear cords are found on the flanks, which were previously considered a pathognomonic sign of IGD. A few cases solely presented papules located on the scalp, neck, as well as on the dorsal surface of the hands and feet. IGD co-occurs in patients with rheumatoid arthritis with relatively high frequency [137]. Histopathological examination typically shows interstitial histiocytic and granulomatous infiltration of the dermis. Slightly rarer features are collagen degeneration and the presence of eosinophilia. However, necrotizing vasculitis is generally not found [138].

### 5.4. Papulonodular Mucinosis

Deposition of mucin in the dermis within lupus lesions or unchanged skin is primarily observed in the upper trunk, arms and face, but other areas of the body may also be involved. The clinical picture is dominated by asymptomatic papules and nodules. This rare condition shows positive correlation with LE activity and renal involvement [1,25].

### 5.5. Calcinosis Cutis

Dystrophic calcification is a rare cutaneous manifestation of SLE. Irregularly distributed indurated nodules are located in the subcutaneous tissue of the extremities and buttocks in women, but there are also very few case reports of facial calcinosis cutis. Incision of the nodule may be accompanied by the evacuation of white-yellow discharge. Calcinosis develops approximately 20 years after SLE diagnosis. However, in the case of lupus panniculitis, it is only 5 years [25,139]. 

### 5.6. Pigmentary Abnormalities

Skin pigmentation abnormalities may be induced by antimalarial drugs used as standard treatment in SLE. Clinically, gray discoloration of the beard, hair and eyelashes is observed, as well as hyperpigmentation of the edges of the nail plates [25].

Mepacrine is an antimalarial and anti-inflammatory drug, which was used before the discovery of chloroquine and hydroxychloroquine. It can be used when conventional LE treatment is ineffective. Nevertheless, mepacrine has few harmless side effects, such as liver enzymes elevation accompanied by yellow discoloration of the skin and conjunctiva. It affects about one third of patients and usually resolves after discontinuation of the drug [140,141].

### 5.7. Nail Abnormalities

The following may form within and around the nail plate: hemorrhagic splinters, nail pitting, ridging, leukonychia, onycholysis, digital clubbing, red lunula, nail dyschromia, nail fold erythema, nail fold hyperkeratosis, ragged cuticles and pterygium inversum unguis [25,142].

### 5.8. Rheumatoid Nodules

Subcutaneous rheumatoid nodules occur in the SLE population with a frequency of only 5–10%. They are usually located in the area of the hands and elbows. Nodules located in the fingers may be accompanied by slight swelling of interphalangeal joints. The differential diagnosis includes granuloma annulare, nodules in necrobiosis lipoidica and xanthogranulomatous necrosis [25,143].

### 5.9. Other Manifestations

Other rare disorders associated with LE include: erythema elevatum diutinum, pyoderma gangrenosum, Sweet’s syndrome, amicrobial pustulosis of the folds, erythema multiforme, seborrheic dermatitis, factitious dermatitis, exanthema, eruptive dermatofibromas, atrophie blanche, Degos-like lesions and acanthosis nigricans [2,25]. 

In addition to the cutaneous manifestations of lupus, it is also worth mentioning the importance of skin race. For example, the black race is predisposed to Libman–Sacks endocarditis and cardiovascular events [144,145]. Moreover, African Americans have an increased prevalence of neurologic manifestations [146], whereas Hispanic and Asian patients have a higher risk of renal, hematologic and multi-organ complications [147]. 

## 6. Conclusions

Cutaneous manifestations may be one of a broad spectrum of SLE symptoms or may occur independently. Due to the fact that SLE is a systemic disease that disturbs the functions of multiple organs, knowledge of its diverse morphological presentations among physicians of various specialties is extremely important. Different types of CLE also bring specific implications of varying severity, such as DLE-induced scarring alopecia and irreversible lipoatrophy in LEP. Consequently, certain skin lesions have not only diagnostic but also prognostic value [39]. Various pathomechanisms underlying the skin lesions induce differences in the clinical picture of the skin. Therefore, a thorough knowledge of the cutaneous manifestations of CLE subtypes can be useful in choosing the appropriate treatment. For instance, classic antibody/T-cell-mediated tissue damage in malar rash responds to antimalarial drugs, thrombotic tissue damage in microangiopathic lupus vasculopathy responds to antiplatelets or anticoagulants, while neutrophil-mediated injury, as in Sweet’s syndrome or pyoderma gangrenosum, responds to dapsone. Considering the above, the morphology of skin lesions is also of therapeutic importance [39]. Different variants of CLE can imitate other dermatoses; therefore, it is very important to be conscious of the clinical picture of rarer forms and often to support the diagnosis by histopathological examination. Especially in CCLE, the course is often recurrent with exacerbations and remissions, so prompt diagnosis and effective treatment are important to prevent persistent scars, which cause permanent tissue deformations and significantly reduce the quality of life of patients. Cooperation between general practitioners, dermatologists, rheumatologists, nephrologists and pathologists in the diagnosis, treatment and monitoring of different variants of LE ensures a high standard of health care and effectiveness of the therapy.

## Data Availability

Data sharing is not applicable.
